# Polymorphisms in Inflammatory Genes Modulate Clinical Complications in Patients With Sickle Cell Disease

**DOI:** 10.3389/fimmu.2020.02041

**Published:** 2020-09-04

**Authors:** Karina Tozatto-Maio, Robert Girot, Indou Deme Ly, Ana Cristina Silva Pinto, Vanderson Rocha, Francisco Fernandes, Ibrahima Diagne, Yahia Benzerara, Carla L. Dinardo, Julia Pavan Soler, Simone Kashima, Itauá Leston Araujo, Chantal Kenzey, Guilherme H. H. Fonseca, Evandra S. Rodrigues, Fernanda Volt, Luciana Jarduli, Annalisa Ruggeri, Christina Mariaselvam, Sandra F. M. Gualandro, Hanadi Rafii, Barbara Cappelli, Felipe Melo Nogueira, Graziana Maria Scigliuolo, Renato Luiz Guerino-Cunha, Kelen Cristina Ribeiro Malmegrim, Belinda P. Simões, Eliane Gluckman, Ryad Tamouza

**Affiliations:** ^1^Eurocord, Université de Paris, IRSL, Hopital Saint Louis, Paris, France; ^2^Monacord, International Observatory on Sickle Cell Disease, Centre Scientifique de Monaco, Monaco, Monaco; ^3^Faculdade de Medicina de Ribeirão Preto, Universidade de São Paulo, Ribeirão Preto, Brazil; ^4^Disciplina de Hematologia e Hemoterapia, Faculdade de Medicina, Universidade de São Paulo, São Paulo, Brazil; ^5^CHU Tenon, Paris, France; ^6^National Children Hospital Center Albert Royer, Cheikh Anta Diop University, Dakar, Senegal; ^7^Center for Cell-based Therapy, Regional Blood Center of Ribeirão Preto, University of São Paulo, Ribeirão Preto, Brazil; ^8^Instituto de Matematica e Estatistica da Universidade de São Paulo, São Paulo, Brazil; ^9^Département de Bactériologie, Hôpital Saint-Antoine, Hôpitaux de l'Est parisien, Paris, France; ^10^INSERM 1160, Université Paris 7, Paris, France; ^11^School of Pharmaceutical Sciences of Ribeirão Preto, University of São Paulo, Ribeirão Preto, Brazil; ^12^Haematology and Bone Marrow Transplant Unit, IRCCS San Raffaele Scientific Institute, Milan, Italy; ^13^Cellular Therapy and Immunobiology Working Party, The European Society for Blood and Marrow Transplantation, Paris, France; ^14^INSERM U955, CHU Henri Mondor, Créteil, France

**Keywords:** sickle cell retinopathy, sickle cell disease, inflammation markers, NK cell receptors and ligands, toll-like receptor (TLR), non-classical HLA, CTLA 4, sickle cell complications

## Abstract

Sickle cell disease (SCD), the most common monogenic disease worldwide, is marked by a phenotypic variability that is, to date, only partially understood. Because inflammation plays a major role in SCD pathophysiology, we hypothesized that single nucleotide polymorphisms (SNP) in genes encoding functionally important inflammatory proteins might modulate the occurrence of SCD complications. We assessed the association between 20 SNPs in genes encoding Toll-like receptors (TLR), NK cell receptors (NKG), histocompatibility leukocyte antigens (HLA), major histocompatibility complex class I polypeptide-related sequence A (MICA) and cytotoxic T-lymphocyte-associated antigen 4 (CTLA-4), and the occurrence of six SCD clinical complications (stroke, acute chest syndrome (ACS), leg ulcers, cholelithiasis, osteonecrosis, or retinopathy). This study was performed in a cohort of 500 patients. We found that the *TLR2 rs*4696480 *TA, TLR2 rs*3804099 *CC*, and HLA-G, *rs*9380142 *AA* genotypes were more frequent in patients who had fewer complications. Also, in logistic regression, the HLA-G *rs*9380142 *G* allele increased the risk of cholelithiasis (*AG* vs. *AA*, OR 1.57, 95%CI 1.16–2.15; *GG* vs. *AA*, OR 2.47, 95%CI 1.34–4.64; *P* = 0.02). For SNPs located in the *NKG2D* loci, in logistic regression, the A allele in three SNPs was associated with a lower frequency of retinopathy, namely, *rs*2246809 (*AA* vs. *GG*: OR 0.22, 95%CI 0.09–0.50; *AG* vs. *GG*: OR 0.47, 95%CI 0.31–0.71; *P* = 0.004, for patients of same origin), *rs*2617160 (*AT* vs. *TT*: OR 0.67, 95%CI 0.48–0.92; *AA* vs. *TT*: OR 0.45, 95%CI 0.23–0.84; *P* = 0.04), and *rs*2617169 (*AA* vs. *TT*: OR 0.33, 95%CI 0.13–0.82; *AT* vs. *TT*: OR 0.58, 95%CI 0.36–0.91, *P* = 0.049, in patients of same SCD genotype). These results, by uncovering susceptibility to, or protection against SCD complications, might contribute to a better understanding of the inflammatory pathways involved in SCD manifestations and to pave the way for the discovery of biomarkers that predict disease severity, which would improve SCD management.

## Background

Sickle cell disease (SCD) is the most common monogenic disorder (1), caused by a single nucleotide polymorphism (SNP) in the first exon of the β-globin gene (*HBB*). The β^S^ allele emerged in some regions of Sub-Saharan Africa and Asia and SCD was initially restricted, mainly, to these areas. However, due to migration flows, the high morbidity and mortality and the global burden associated with the disease, SCD became a worldwide public health issue (2). Patients with SCD present several acute and chronic complications, some of them life-threatening. However, to date, with very few exceptions, such as stroke in children (3), most complications cannot be predicted.

A hallmark of SCD is the phenotype variability that occurs even within the same SCD genotype (SS, Sβ, SC), which is not fully understood (2). SCD complications occur due to vaso-occlusion, hemolysis due to red cell injury, and inflammation, resulting from various triggers, including intravascular hemolysis and ischemia-reperfusion injury (4–6). Although inflammation plays a major role in the pathophysiology of the disease, not all inflammatory pathways involved in SCD complications are known.

Some studies have addressed the influence of inflammatory markers in SCD complications, but few of them have evaluated the role of polymorphisms in genes encoding pivotal inflammatory proteins belonging to both innate and adaptive immune processes in the occurrence of complications (7–16). We hypothesize that such genes can modulate the occurrence of some complications in patients with SCD. Finding clinical and biological markers for predicting SCD complications would help to prevent them, and to decrease morbidity and mortality. Our work aimed to find associations between specific complications in patients with SCD and relevant polymorphisms in genes encoding potent inflammatory molecules.

## Methods

### Study Population

This was a case-control retrospective study. Five hundred patients, followed at the Albert Royer Hospital, Dakar, Senegal (*n* = 56), Clinics Hospital of Ribeirão Preto, Ribeirão Preto, Brazil (*n* = 142), Clinics Hospital of São Paulo, São Paulo, Brazil (*n* = 88), and Tenon Hospital, Paris, France (*n* = 214), mainly from Sub-Saharan Africa and at a lesser extent from the French West Indies were included in the study. All patients had been previously diagnosed with SCD by hemoglobin electrophoresis or high-performance liquid chromatography (HPLC), had clinical records available for data collection and stored DNA samples.

We analyzed the potential influence of 20 SNPs on the occurrence of six clinical complications: acute chest syndrome (ACS), stroke, leg ulcers, cholelithiasis, osteonecrosis, and sickle retinopathy of any grade, occurring at any time from birth to last follow up. For each complication, a case was defined as a patient who had the specific complication, and a control was defined as a patient, from the same study cohort, who did not present the complication. ACS was defined as the presence of an acute respiratory symptom and a new pulmonary consolidation shown on chest radiography (17, 18). Stroke was defined as a new finding in neurological exam and a new image on brain computed tomography (CT). Cholelithiasis was documented by abdominal ultrasonography or CT. Osteonecrosis of any bone involving or not joint was documented by radiography, CT or magnetic resonance imaging (MRI). Retinopathy was documented by examination performed by an ophthalmologist; because the pathophysiology bases are the same, both non-proliferative and proliferative retinopathy were included.

### Genotyping

DNA was extracted from peripheral blood samples using standard methods (19). Using TaqMan 5′-nuclease assay (Applied Biosystems, Forster City, CA, USA) pre-designed allele-specific fluorogenic oligonucleotide probes, we genotyped SNPs in genes encoding the following innate and adaptative immune molecules: Toll-like receptor (TLR)1 (*rs*4833095), TLR2 (*rs*4308099, *rs*4308100, *rs*4696480), TLR6 (*rs*5743810), TLR10 (*rs*11466653, *rs*11096957), all located in chromosome 4 and belonging to the TLR1 subfamily (20); natural killer (NK) group 2 member D (NKG2D) receptor (*rs*1982536, *rs*2617160, *rs*2617169, *rs*2617170, *rs*2617171, *rs*1049174, *rs*2246809, *rs*2255336), in chromosome 12; human leukocyte antigen (HLA)-G (*rs*9380142), HLA-E (*rs*2517523), major histocompatibility complex class I polypeptide-related sequence A (MICA, *rs*1057192), in the chromosome 6; and cytotoxic T-lymphocyte-associated antigen 4 (CTLA-4) (*rs*5742909, *rs*231775), in the chromosome 2. DNA pre-amplification and real-time polymerase chain reaction (RT-PCR) were performed as previously described (15, 21). All SNPs included in this analysis were selected based on the relevance of their biological function.

### Comparison With Non-SCD Populations

For all SNPs, we compared allele distribution between the study population and the African population described on the 1,000 genomes database (22), composed by 661 healthy adult individuals, mainly from Sub-Saharan African and African-American origin. This was an exploratory comparison to determine if the frequency of risk alleles was the same in SCD and non-SCD individuals. For some SNPs, we also performed comparisons with a Brazilian non-SCD population, composed of 138 adult healthy individuals.

### Statistical Analysis

We performed analyses of correspondence, represented by dot charts, for indicating associations between SNPs and number of clinical complications; the significance of the association was tested using odds ratio (OR). Using item response theory (IRT) (23, 24), we also calculated a score for each patient based on the occurrence of each clinical complication; based on this score, we built characteristic curves for each clinical complication in the study population. Associations between SNPs and each complication were assessed by logistic regression; for modeling adjustment, geographical origin, SCD genotype, rate of red blood cell (RBC) transfusions (higher rate of transfusions indicating more severe disease) and gender were considered, and the significance was adjusted for multiple testing using false discovery rate (FDR). For all SNPs, two genetic models were used: the additive model, which supposes that the effect of the risk allele increases with the addition of each allele copy; and the additive-dominant model, which analyzes the effect of the heterozygous genotype vs. homozygotes. Analyses were performed using R software, version 3.4.4 (25). R: A language and environment for statistical computing. R Foundation for Statistical Computing, Vienna, Austria. ISBN 3-900051-07-0, http://cran.r-project.org/doc/manuals/R-intro.pdf).

### Ethical Considerations

The study was approved by internal review boards, by the Eurocord scientific committee and was performed according to the declaration of Helsinki.

## Results

### Demography

Five hundred patients were included in the study, originally from Brazil (*n* = 228), Sub-Saharan Africa (*n* = 200), French West Indies (*n* = 53), and North Africa (*n* = 7); 12 patients did not have their geographical origin reported. SCD genotype was available for 494 patients and was mostly SS (*n* = 402), followed by SC (*n* = 46), Sβ (*n* = 42), or other (*n* = 4). Two hundred and eighty patients were female and median age of the whole cohort was 32 (range: 0–69 years).

### Clinical Complications

Ninety-seven (19%) patients did not present any of the six clinical complications analyzed in this study; among patients who presented complications, 135 (27%) had one, 134 (27%) had two, 94 (19%) had three, 34 (7%) had four, and 6 (1%) had five types of complications, respectively.

**Figure 1:**
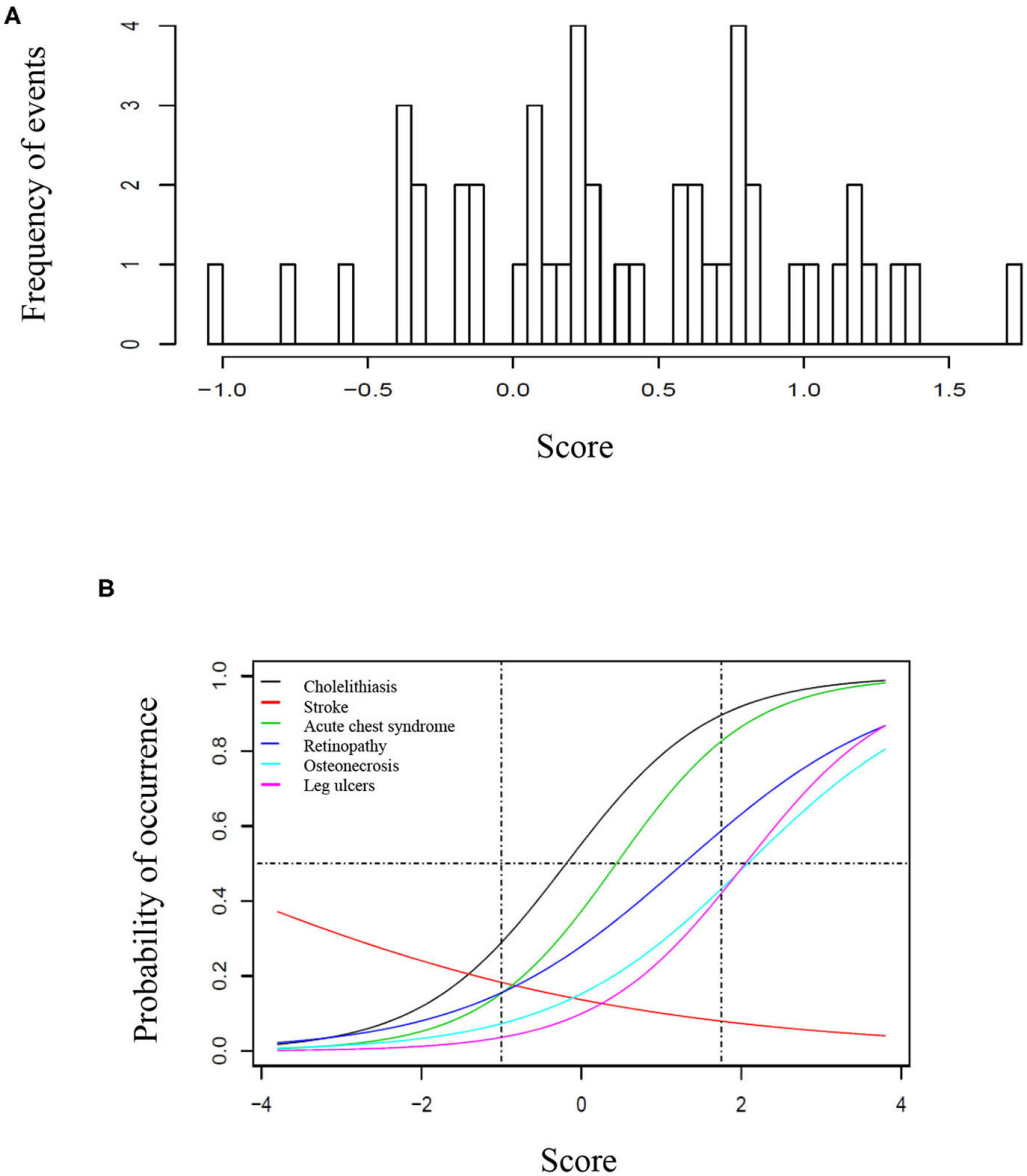
Score of clinical complications in the study cohort. **(A)** Histogram representing the score of clinical complication calculated for each patient based on the item-response theory (ITR). **(B)** Characteristic curves for each clinical complication, based on the IRT score. **(A)** Represents the score based on the distribution of complication for each patient, ranging from −1 to ~1, 8. In **(B)**, the curves are shown from −4 to 4 to better illustrate the trend of each curve, however the vertical traced lines indicate the interval where patients are located. In this cohort, the probability of occurrence was higher for cholelithiasis, followed by acute chest syndrome, retinopathy, osteonecrosis, and leg ulcers. The horizontal line represents 50% of probability. For instance, a patient with 50% probability of having retinopathy had >50% probability of presenting cholelithiasis and acute chest syndrome, but <50% for osteonecrosis and leg ulcers. Patients who presented stroke had less probability of presenting all other clinical complications.

Seventy-one/500 patients (14%) presented at least one episode of stroke, 200/500 (40%) had at least one episode of ACS, 69/500 (14%) had leg ulcers, 271/500 (54%) presented cholelithiasis, 150/366 (41%) had retinopathy, and 90/500 (18%) presented osteonecrosis. For retinopathy, we have only considered patients who had retinal screening available in medical records. One hundred eighty-four patients (37%) received at least 20 RBC transfusions. Twenty-one (4%) patients underwent hematopoietic stem cell transplantation. Eleven (2%) patients died during follow-up, mostly from acute chest syndrome and hemorrhagic stroke. Table 1 shows the frequency of clinical complications according to gender, origin and SCD genotype. Figure 1A shows the score calculated for each patient and Figure 1B represents the curves of distribution of how many clinical complications were observed in this cohort. Patients who had stroke showed less probability of presenting other complications.

**Table 1:** Demography according to clinical complications.

		**Age**	**Gender (*****n*** **=** **498)**		**Origin (*****n*** **=** **488)**				**SCD genotype (*****n*** **=** **496)**	
	***n***	**Median age (range)**	**Female**	**Male**	**Brazilian**	**Sub-Saharan Africa**	**French West Indies**	**North Africa**	**SS/Sβ/other**	**SC**
General population	500	32 (0–69)	280	218	228	200	53	7	448	46
ACS (*n* = 500)										
No	300	31 (0–69)	165	133	144	84	15	1	264	33
Yes	200	32 (2–59)	115	85	84	78	31	4	184	13
Stroke (*n* = 500)										
No	429	32 (0–69)	246	181	162	155	41	5	377	46
Yes	71	24 (7–55)	34	37	66	3	3	0	71	0
Leg ulcers (*n* = 500)										
No	431	31(0–69)	247	182	189	127	31	5	380	46
Yes	69	39 (22–64)	33	36	39	19	10	0	68	0
Cholelithiasis (*n* = 500)										
No	229	31 (0–60)	110	117	72	70	9	0	193	33
Yes	271	32 (8–69)	170	101	156	77	29	4	255	13
Retinopathy (*n* = 366)										
No	216	27 (3–69)	130	86	138	64	7	5	207	5
Yes	150	37 (12–69)	87	63	26	85	35	0	114	35
Osteonecrosis (*n* = 500)										
No	410	31 (0–69)	228	180	200	90	25	1	366	39
Yes	90	34 (13–58)	52	38	28	44	12	3	82	7

### Genetic Analyses

All SNPs had a call rate >90% and minimum allele frequency >1%. *TLR2 rs*4696480, *TLR6 rs*5743810, *NKG2D rs*1049174, *NKG2D rs*2617171, and *MICA rs*1051792 SNPs did not satisfy the expected Hardy-Weinberg proportions. However, because our cohort was composed only of patients with sickle cell disease, with no healthy controls, we kept these SNPs for genotype analysis. Genotype distribution of each SNP in our cohort is shown on Table 2. Linkage disequilibrium between SNPs is shown on Figure S1.

**Table 2:** Genotype distribution for each SNP in the study population and according to clinical complication.

	**Total (*n* = 500)**	**Stroke (*****n*** **=** **500)**		**Acute chest syndrome (*****n*** **=** **500)**		**Leg ulcers (*****n*** **=** **500)**		**Osteonecrosis (*****n*** **=** **500)**		**Cholelithiasis (*****n*** **=** **500)**		**Retinopathy (*****n*** **=** **366)**		**Total retinopathy (*n* = 366)**
		**No**	**Yes**	**No**	**Yes**	**No**	**Yes**	**No**	**Yes**	**No**	**Yes**	**No**	**Yes**	
*rs*4696480	495													364
AA	164	131	33	87	77	135	29	136	28	57	107	74	55	129
AT	280	249	31	179	101	247	33	233	47	145	135	120	80	200
TT	51	48	3	30	21	45	6	39	12	27	24	20	15	35
*rs*4833095	470													344
AA	142	124	18	80	62	122	20	116	26	67	75	45	55	100
AG	210	180	30	117	93	186	24	176	34	88	122	90	66	156
GG	118	97	21	81	37	95	23	95	23	53	65	66	22	88
*rs*2246809	495													361
AA	34	30	4	26	8	30	4	27	7	16	18	19	5	24
AG	197	177	20	115	82	178	19	162	35	101	96	91	57	148
GG	264	218	46	155	109	218	46	218	46	111	153	103	86	189
*rs*9380142	487													354
AA	295	256	39	182	113	255	40	245	50	153	142	116	93	209
AG	166	145	21	95	71	144	22	133	33	59	107	79	48	127
GG	26	19	7	16	10	20	6	22	4	11	15	14	4	18
*rs*3804099	496													363
CC	176	159	17	104	72	157	19	144	32	93	83	73	68	141
CT	222	192	30	127	95	192	30	183	39	95	127	99	60	159
TT	98	75	23	65	33	78	20	80	18	39	59	43	20	63
*rs*5743810	497													363
AA	12	7	5	8	4	11	1	12	0	6	6	6	1	7
AG	68	53	15	46	22	61	7	60	8	24	44	42	11	53
GG	417	366	51	245	172	356	61	335	82	197	220	166	137	303
*rs*2617169	497													363
AA	12	12	0	9	3	10	2	10	2	7	5	7	1	8
AT	144	131	13	86	58	130	14	122	22	73	71	70	37	107
TT	341	283	58	202	139	288	53	276	65	148	193	138	110	248
*rs*2517523	484													356
AA	112	96	16	63	49	92	20	96	16	53	59	47	31	78
AG	223	190	33	139	84	196	27	176	47	105	118	103	71	174
GG	149	128	21	86	63	129	20	126	23	61	88	61	43	104
*rs*3804100	485													357
CC	1	1	0	1	0	1	0	1	0	1	0	0	0	0
CT	43	34	9	28	15	37	6	34	9	17	26	188	134	322
TT	441	381	60	258	183	380	61	363	78	201	240	23	12	35
*rs*1049174	496													362
CC	77	53	24	44	33	64	13	65	12	22	55	34	12	46
CG	182	156	26	110	72	158	24	154	28	83	99	89	58	147
GG	237	216	21	144	93	205	32	188	49	123	114	90	79	169
*rs*2617170	499													366
CC	95	82	13	54	41	85	10	76	19	40	55	35	34	69
CT	246	218	28	150	96	209	37	201	45	121	125	114	80	194
TT	158	129	29	95	63	136	22	133	25	68	90	67	36	103
*rs*1051792	473													349
AA	195	162	33	123	72	169	26	159	36	93	102	81	61	142
AG	169	146	23	91	78	149	20	143	26	76	93	73	56	129
GG	109	94	15	63	46	88	21	86	23	38	71	49	29	78
*rs*11466653	496													365
AA	450	392	58	272	178	389	61	369	81	210	240	191	141	332
GA	44	34	10	24	20	37	7	36	8	18	26	23	8	31
GG	2	2	0	1	1	2	0	2	0	1	1	2	0	2
*rs*2255336	498													365
CC	165	128	37	102	63	138	27	140	25	60	105	77	40	117
CT	232	208	24	130	102	202	30	186	46	116	116	99	78	177
TT	101	92	9	66	35	89	12	83	18	53	48	40	31	71
*rs*2617171	485													356
CC	246	225	21	146	100	215	31	197	49	128	118	94	87	181
CG	161	137	24	98	63	139	22	139	22	71	90	82	47	129
GG	78	58	20	45	33	65	13	66	12	25	53	33	13	46
*rs*5742909	470													345
CC	434	376	58	251	183	371	63	353	81	191	243	177	144	321
CT	35	25	10	24	11	30	5	34	1	13	22	21	2	23
TT	1	1	0	0	1	1	0	0	1	1	0	1	0	1
*rs*11096957	496													362
GG	129	115	14	71	58	108	21	105	24	61	68	57	41	98
GT	253	216	37	159	94	220	33	207	46	116	137	108	70	178
TT	114	95	19	67	47	100	14	94	20	52	62	49	37	86
*rs*2617160	496													364
AA	94	85	9	62	32	85	9	76	18	45	49	48	20	68
AT	248	213	35	151	97	213	35	201	47	120	128	117	81	198
TT	154	128	26	83	71	130	24	131	23	63	91	50	48	98
*rs*1983526	496													362
CC	26	19	7	12	14	23	3	23	3	9	17	9	6	15
CG	155	126	29	98	57	128	27	129	26	65	90	74	43	117
GG	315	280	35	186	129	276	39	256	59	154	161	131	99	230
*rs*231775	469													347
AA	177	157	20	106	71	153	24	151	26	82	95	74	58	132
AG	221	182	39	128	93	189	32	172	49	93	128	105	61	166
GG	71	63	8	40	31	59	12	62	9	31	40	23	26	49

*For all SNPs, the total n is <500 because of missing genotyping data*.

### Effect of TLR SNPs on Clinical Complications

**Figure 2:**
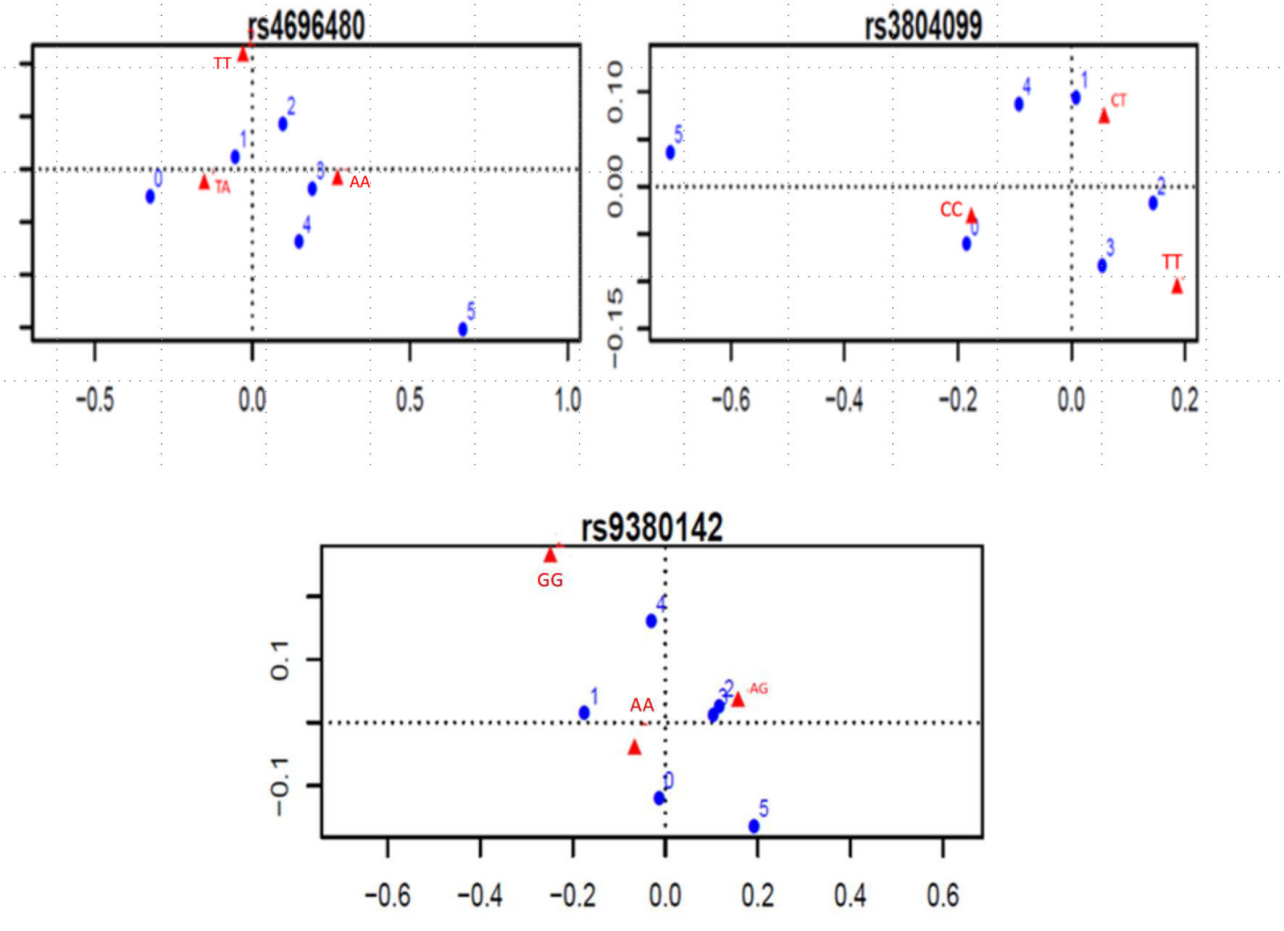
Significant correspondence analysis between SNP genotypes and number of complications. Blue dots represent the number of complications, ranging from 0 to 5. Red triangles represent the genotypes of each SNP.

Considering the indication of associations between SNPs and number of clinical complications, the *TLR2 rs*4696480 *TA* genotype was found more frequently in patients who had no complications or one complication (*TA* vs. *AA and TT*, 53 vs. 38%, OR 0.55, 95%CI 0.38–0.79, *P* = 0.001); The *TLR2 rs*3804099 *CC* genotype occurred more frequently in patients who had no complications (*CC* vs. *CT* or *TT;* 45 vs. 33%, OR 1.62, 95%CI 1.03–2.56, *P* = 0.03). Figure 2 shows the dot charts of the correspondence analysis. By mean of logistic regression models, we found no association between any of the SNPs in *TLR* genes and clinical complications. Table 3 summarizes the results of the logistic regression analyses.

**Table 3:** Significant SNPs, according to the clinical variable, in logistic regression models.

**Clinical complication**	**Genotype**	**Odds ratio**	**95% CI**	***P*-value**
**Cholelithiasis**				
*HLA-G rs*9380142	*AA*	1		0.02
	*AGxAA*	1.57	1.16–2.15	
	*GGxAA*	2.47	1.34–4.64	
**Retinopathy**				
*NKG2D rs*2246809	*GG*	1		0.004
	*AGxGG*	0.47	0.31–0.71	
	*AAxGG*	0.22	0.09–0.50	
*NKG2D rs*2617160	*TT*	1		0.04
	*ATxTT*	0.67	0.48–0.92	
	*AAxTT*	0.45	0.23–0.84	
*NKG2D rs*2617169	*TT*	1		0.049
	*ATxTT*	0.58	0.36–0.91	
	*AAxTT*	0.33	0.13–0.82	

### Effect of NKG2D SNPs on Clinical Complications

Considering the associations between SNPs and each clinical complication, three *NKG2D* SNPs were significantly associated with the occurrence of retinopathy. The distribution of genotypes of these SNPs was: *rs*2246809, retinopathy group (*n* = 148): *GG* 58%, *AG* 39%, *AA* 3%; non-retinopathy group (*n* = 213): *GG* 48%, *AG* 43%, *AA* 9%; *rs*2617160, retinopathy group (*n* = 149): *TT* 32%, *AT* 55%, *AA* 13%; non-retinopathy group (*n* = 215): *TT* 23%, *AT* 55%, *AA* 22%; *rs*2617169, retinopathy group (*n* = 148): *TT* 1%, *AT* 25%, *AA* 74%; non-retinopathy group (*n* = 215): *TT* 3%, *AT* 33%, *AA* 64%. In the logistic regression additive model, the A allele in these SNPs was associated with a lower risk of retinopathy: *rs*2246809, *AA* vs. *GG*: OR 0.22, 95%CI 0.09–0.50; *AG* vs. *GG*: OR 0.47, 95%CI 0.31–0.71, in patients of same origin; *P* = 0.004; *rs*2617160, *AT* vs. *TT*: OR 0.67, 95%CI 0.48–0.92; *AA* vs. *TT*: OR 0.45, 95%CI 0.23–0.84; *P* = 0.04; *rs*2617169, *AA* vs. *TT*: OR 0.33, 95%CI 0.13–0.82; *AT* vs. *TT*: OR 0.58, 95%CI 0.36–0.91, *P* = 0.049, in patients of same SCD genotype. We did not find any association between *NKG2D* SNPs and other clinical complications.

### Effect of HLA-G, HLA-E, and MICA SNPs on Clinical Complications

The presence of the *A* allele for *MICA rs*1051792 (genotypes *AA/AG)* was associated with overall less complications (up to two complications), but this was not statistically significant. *HLA-G rs*9380142 was the only SNP significantly associated with cholelithiasis in the logistic regression additive model. The distribution of genotypes in the cholelithiasis group (*n* = 264) was: *AA* 54%, *AG* 40%, *GG* 6%, and in the non-cholelithiasis group (*n* = 223), *AA* 69%, *AG* 26%, and *GG* 5%. The *G* allele increased the risk of cholelithiasis (*AG* vs. *AA*, OR 1.57, 95%CI 1.16–2.15; *GG* vs. *AA*, OR 2.47, 95%CI 1.34–4.64; *P* = 0.02). In addition, the *AA* genotype of this SNP was significantly more frequent in patients who had 0–1 complication than genotypes AG and GG (65 vs. 56%, OR 1.47, 95%CI 1.02–2.12, *P* = 0.03). Furthermore, although MICA is a ligand for HLA-E, we did not find any significant association between the SNPs in the corresponding genes in this cohort.

### Effect of CTLA4 SNPs on Clinical Complications

No association was found between alleles or genotypes in the SNPs encoding CTLA4 and clinical manifestations of SCD.

### Effect of Haplotypes on Clinical Complications

No haplotype in this analysis was associated with complications.

### Comparison With Non-SCD Populations

Figure S2 shows a graph representing the allele distribution of SNPs in our SCD cohort and in two non-SCD populations, the one described at the 1,000 genomes database, and a previously studied Brazilian population composed by non-SCD patients (not available for all SNPs).

## Discussion

Patients with SCD have a steady state inflammatory background that can be boosted by ischemia-reperfusion injury, hemolysis and other situations such as infections (4). In addition, SCD has a marked phenotype variability not fully understood. It was previously demonstrated that polymorphisms in genes encoding inflammatory proteins play a role in the susceptibility to some complications in patients with SCD (7–16, 26).

NKG2D is a transmembrane receptor expressed on the surface of NK cells, iNKT cells, T CD8+ and some subsets of TCD4+ lymphocytes (27). NKG2D is encoded by a gene located in the natural killer complex (NKC) gene region on chromosome 12 and belong to the NK cell activating receptors. For instance, upon interaction with its ligand on the target cell, NK cell exert their cytotoxic functions and upregulate IFN-gamma secretion with consequent naïve cytotoxic T lymphocytes in CD8+ alpha/beta T cells activation, thus helping in the elimination of triggering event including, for example, tumor cells or pathogens (28–32). NKG2D recognizes a wide range of ligands, such as MICA and UL16-binding proteins (ULBP). However, NKG2D ligands are not spontaneously expressed but are induced upon stimulation, for example stress, viral or bacterial infections (27). Any type of cell can express NKG2D ligands when stimulated, and dysregulation of NKG2D and its ligands was observed in cancer and in autoimmune diseases such as rheumatoid arthritis and alopecia areata (30, 33, 34).

Patients with SCD have higher levels of circulating leukocytes (4, 35); higher levels of NKT cells/activated NKT cells in this population, compared with non-SCD controls, were shown in a study (36). In SCD, ischemia-reperfusion injury due to repeated episodes of vaso-occlusion triggers inflammation by activation of CD1d-restricted iNKT cells (36). NKG2D acts as a costimulatory factor for iNKT activation in response to CD1d and as an independent factor for iNKT activation (37). Haplotypes composed by SNPs scattered along the NKC region, especially NKG2D, were previously associated with high or low natural cytotoxic activity in cancer settings (38). Thus, we tested these SNPs because they have a defined role in NKG2D function. We found that three SNPs in *NKG2D* influence the occurrence of retinopathy. For these three SNPs, the A allele decreased the risk of retinopathy. In all cases, previously, the A allele was associated with a higher potential of cellular cytotoxicity likely in relation with enhanced inflammatory processes in the functional haplotypes studied by Hayashi et al. (38).

Retinopathy is the most prevalent vascular complication in SCD (39). It occurs in up to 46% of SS patients and 76% of SC patients (39–41). Proliferative retinopathy also occurs more in SC patients than in SS (42); it was proposed that the more severe phenotype of SS patients might lead to early occlusion of the peripheral retinal vessels, thus impairing neovascularization, whereas SC patients, because of their milder phenotype, would have retinal vessels preserved for a longer time, making neovascularization possible in this context (41). We hypothesize that patients with higher inflammatory profile might have had more severe occlusion during early years of life, leading to less neovascularization and clinically less retinopathy. Nevertheless, a recent study found that use of hydroxyurea, with HbF levels >15%, is associated with less retinopathy in SS patients (39). Hydroxyurea, besides increasing HbF expression, lowers the levels of circulating leukocytes and has anti-inflammatory effects in SCD (43, 44). Prospective studies assessing inflammatory markers and retinopathy in these settings are warranted for confirming our hypothesis.

MICA, a transmembrane glycoprotein encoded by the highly polymorphic *MICA* locus, located in the major histocompatibility complex (MHC) in chromosome 6. *MICA*, the most polymorphic non-classical HLA-class I gene, is expressed in few specific cell types upon cellular and genotoxic stress, malignant transformation, and viral infection [reviewed in Isernhagen et al. (45)]. MICA acts as a ligand for NKG2D. The SNP *rs*1059172 is of interest, because a missense mutation in this position (position 129 of the MICA protein, *G* to *A*) leads to an amino-acid change, from valine to methionine. The isoform with methionine has high affinity for NKG2D, resulting in enhanced NKG2D signaling, whereas valine determines low affinity (46, 47). Nevertheless, in our study, the A allele was associated with less complications, which is somewhat contra-intuitive. Isernhagen et al. (47) reviewed studies of association between *rs*1051792 and several diseases or clinical complications; the association of alleles and genotypes with higher or lower inflammatory responses was not consistent. Therefore, for this SNP, although the role of the amino-acid change in NKG2D activation is biologically established, its clinical repercussion in several disease settings, including SCD, needs further evaluation. One possible hypothesis, given that the main function of NK cells is the pivotal cellular cytotoxicity mediated anti-infectious responses, is that overall, the observed association could reflect a protective effect against infections acting before the occurrence of inflammatory-mediated complications.

HLA-G is involved in immunotolerance and, in physiological conditions, is expressed in some restricted human tissues and in the plasma; altered regulation of HLA-G expression has been described in several inflammatory diseases (48). Nevertheless, few studies investigated the role of HLA-G in SCD. In our study, the SNP *rs*9380142 *AA* genotype was associated with less complications, whereas the *G* allele was associated with more cholelithiasis. Cholelithiasis in SCD occurs due to hemolysis, which contributes to inflammatory activation in SCD settings (6). Thus, we hypothesize that the association found might reflect higher inflammatory activation because of hemolysis in patients carrying the *G* allele. Nevertheless, to date, there is no association between HLA-G and hemolysis in SCD. A study evaluating the association of SNPs in the HLA-G locus and the occurrence of hepatitis C virus (HCV) infection showed no effect of HLA-G SNPs in hemoglobin, lactate dehydrogenase and bilirubin levels (49). In our study, we did not have laboratory parameters of hemolysis for further comparisons.

In this cohort, most patients presented at least two SCD complications. Patients who have more complications often show a more severe phenotype, including life-threatening complications. Therefore, finding markers that predict the amount of complications is of value, because anticipating disease severity might lead to treatment optimization. Of note, the TLR2 *rs*4696480 *TA* genotype was significantly associated with occurrence of 0–1 clinical complications. Previously, our group has found that the same genotype was associated with less bacterial infections in SCD (15). Patients with SCD exhibit a marked susceptibility to infections, compared with non-SCD individuals, due to several factors, such as loss of spleen function and the chronic inflammatory status (50). This explains why comparisons between SCD and non-SCD populations, and among SCD patients, may uncover different effects of a given SNP—for instance, alleles and genotypes that modify the function of TLR and modulate the occurrence of infections in this population. Based on our findings and in previous studies on the functional role of this SNP (15, 51–56), we hypothesized that *TA* genotype might determine a balanced inflammatory response, whereas the *TT* genotype would determine a weak inflammatory response, and the *AA* genotype, an overwhelming inflammatory response pattern. Further studies assessing the role of this SNP and its protein expression are required in SCD settings.

Interestingly, patients who had stroke were less likely to show other complications. Life-long chronic transfusion therapy is well-established as long-term secondary prophylaxis for stroke. Thus, the lower probability of other complications in this set is probably explained by the higher rate of patients receiving chronic transfusion to lower HbS.

The retrospective nature of this study prevented us to further investigate causal relations between the SNPs and SCD complications. Furthermore, for the same reason, it was not possible to further assess the mechanisms involved in the modulation of inflammatory response promoted by the SNPs that had significant associations with SCD complications. Also, we cannot exclude that the associations found in this study might reflect the effect of SNPs in other genes inherited in LD with the SNPs tested. However, the size of the cohort improves the strength of our findings. Moreover, we have chosen SNPs that had a previously demonstrated role in inflammation. Studies including larger and/or prospective cohorts, assessing genome-wide associations and gene expression are warranted to further elucidate the influence of different inflammatory pathways in SCD pathophysiology. In addition, because the steady-state inflammatory status found in this disease, which may lower the threshold for activating some inflammatory pathways, SCD might be a good model for studying inflammatory genes.

The recent understanding about inflammation in SCD led to the development of drugs that block inflammatory pathways (57, 58). However, specific inflammatory pathways that determine the occurrence of some complications remain unknown; in addition, it is worrisome that we still cannot predict the occurrence of most complications in a disease with such a heterogeneous clinical presentation. A greater knowledge on inflammatory drivers of SCD would help not only finding biomarkers for predicting complications, but also further developing targeted therapies. Furthermore, establishing a genetic inflammatory profile of patients with severe SCD might allow the identification of patient sub-groups potentially eligible for curative hematopoietic stem cell transplantation or gene therapy.

## Data Availability Statement

The raw data supporting the conclusions of this article will be made available by the authors, without undue reservation, to any qualified researcher.

## Ethics Statement

The studies involving human participants were reviewed and approved by Comitê de Etica em Pesquisa, Faculdade de Medicina de Ribeirão Preto, Universidade de São Paulo; Eurocord scientific committee. Written informed consent to participate in this study was provided by the participants' legal guardian/next of kin.

## Author Contributions

KT-M, BS, EG, RG, and RT designed the study. KT-M, IL, and CK collected clinical data. KT-M performed experiments. CM and IA advised experiments. FF, JS, and KT-M performed statistical analysis. RG, YB, ID, IL, AS, SK, ER, SG, GF, VR, FN, RC, CD, KM, and LJ provided cases and samples for the study. KT-M, FV, AR, EG, BS, RG, HR, BC, GS, and RT wrote the manuscript. All authors edited and approved the manuscript.

## Conflict of Interest

The authors declare that the research was conducted in the absence of any commercial or financial relationships that could be construed as a potential conflict of interest.

## Supplementary Material

Figure S1 Linkage disequilibrium between SNPs. **(A)** Linkage disequilibrium between *TLR* SNPs. **(B)** Linkage disequilibrium between *NKG* SNPs. **(C)** Linkage disequilibrium between *HLA, MICA*, and *CTLA4* SNPs. Linkage disequilibrium (LD) is shown on heat maps. The darker the color, the higher the LD presented between SNPs.

Figure S2 Comparison of the allele distribution of each SNP between our cohort and non-SCD populations. 1000G, 1,000 genomes database; BRZ, Brazilian non-SCD population.
