# Supplementary figures and images for: Polymorphisms in Inflammatory Genes Modulate Clinical Complications in Patients With Sickle Cell Disease
Source: Front Immunol. 2020 Sep 4;11:2041. doi: 10.3389/fimmu.2020.02041 (PMC7510050; doi:10.3389/fimmu.2020.02041)

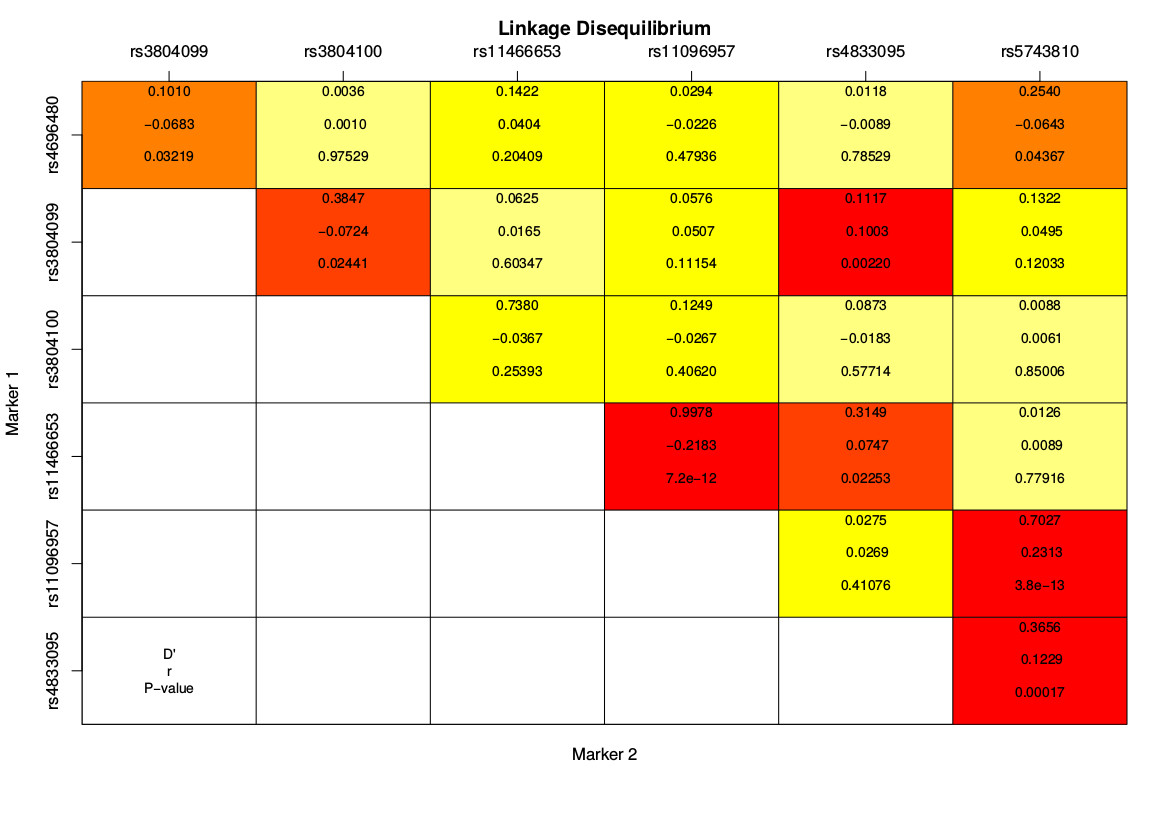

Supplement: Supplementary file 1 [file Image_1.jpg]

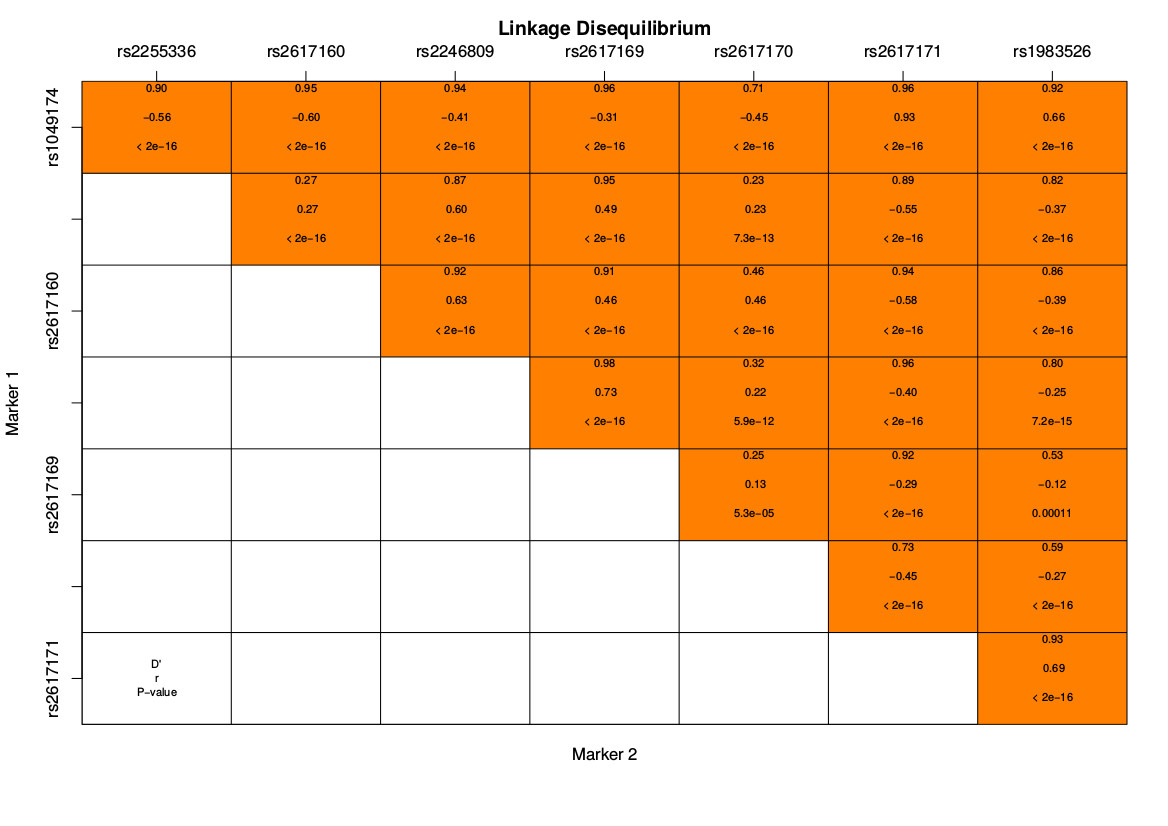

Supplement: Supplementary file 2 [file Image_2.jpg]

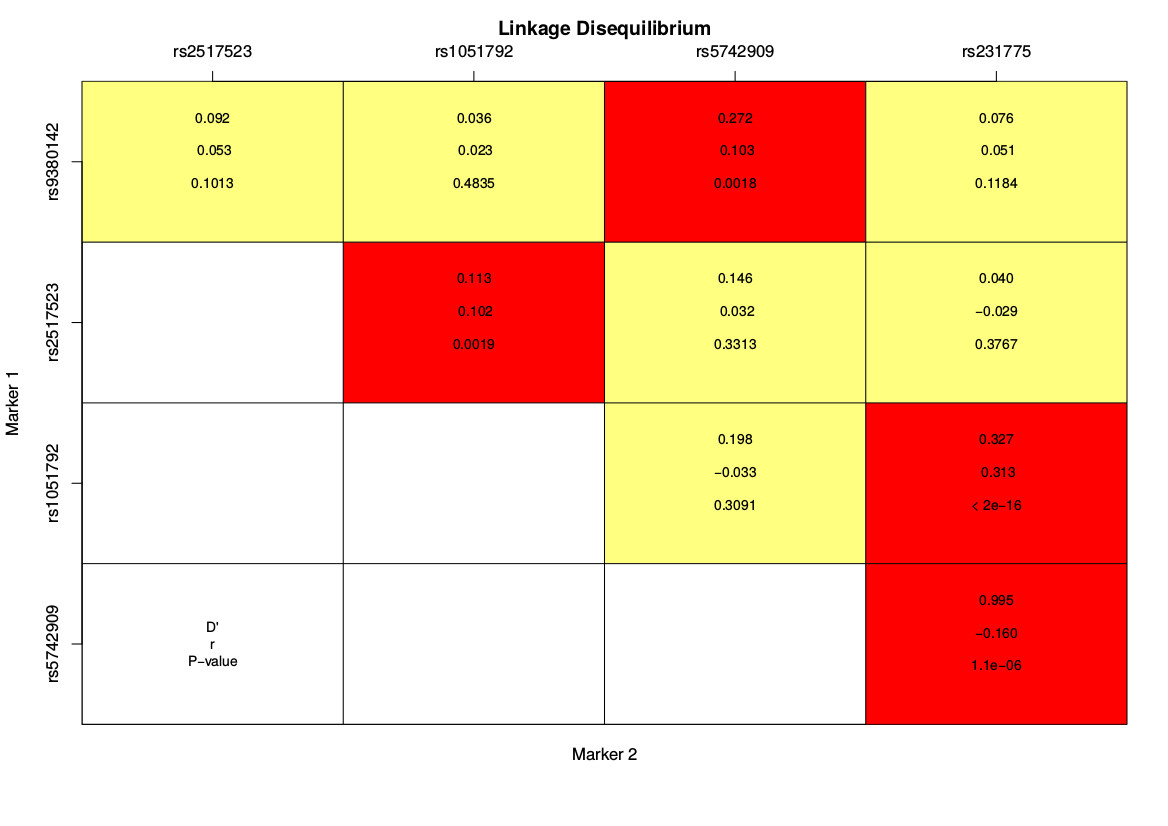

Supplement: Figure S1 — Linkage disequilibrium between SNPs. (A) Linkage disequilibrium between TLR SNPs. (B) Linkage disequilibrium between NKG SNPs. (C) Linkage disequilibrium between HLA, MICA, and CTLA4 SNPs. Linkage disequilibrium (LD) is shown on heat maps. The darker the color, the higher the LD presented between SNPs. [file Image_3.jpg]

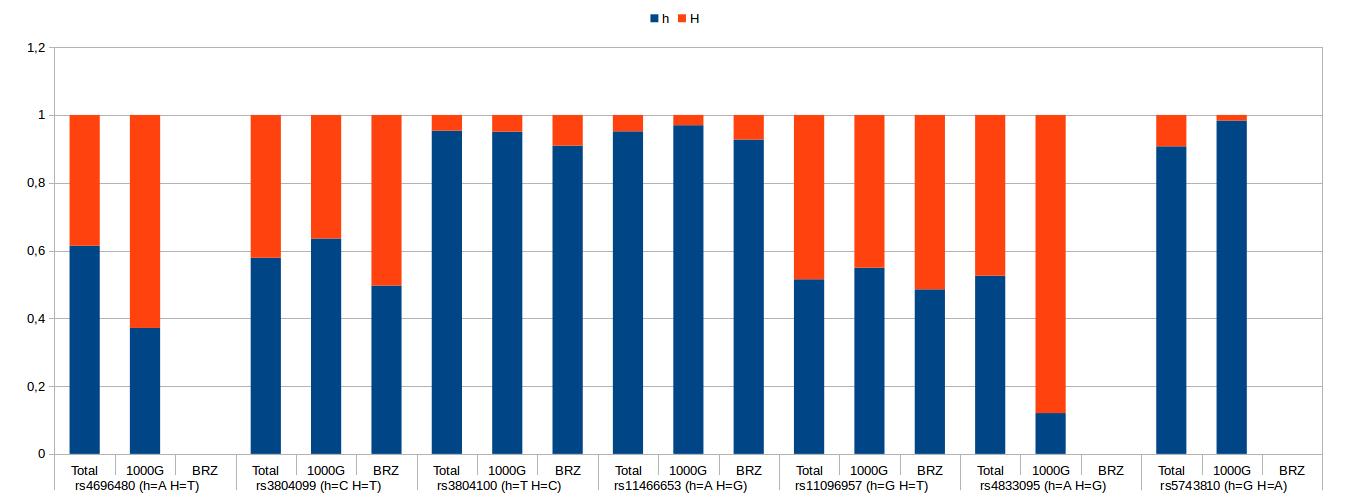

Supplement: Supplementary file 4 [file Image_4.jpg]

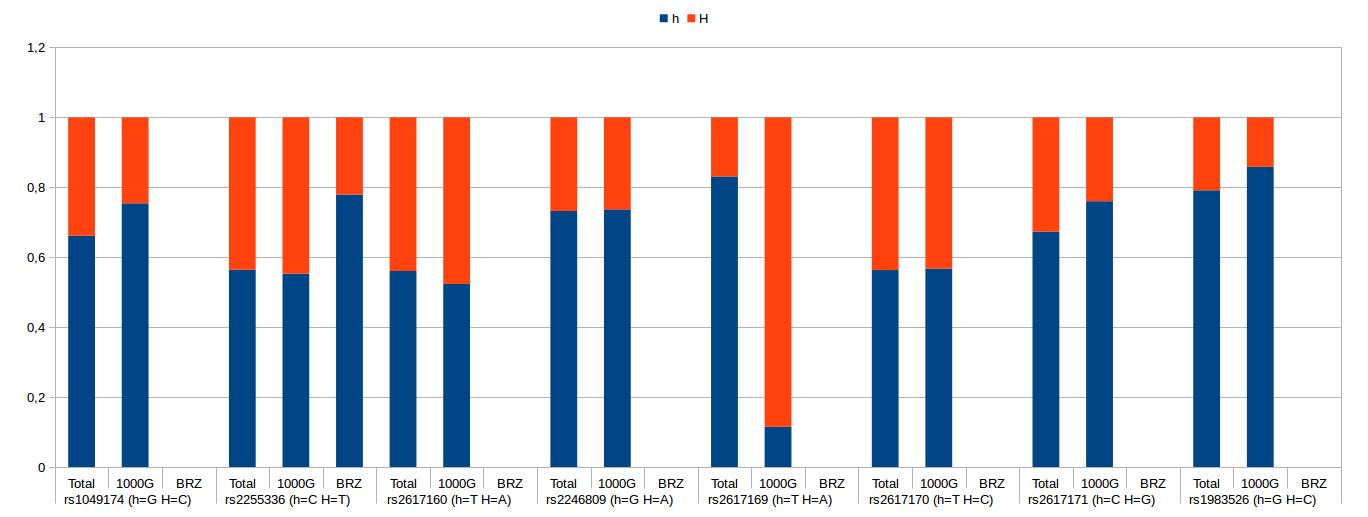

Supplement: Supplementary file 5 [file Image_5.jpg]

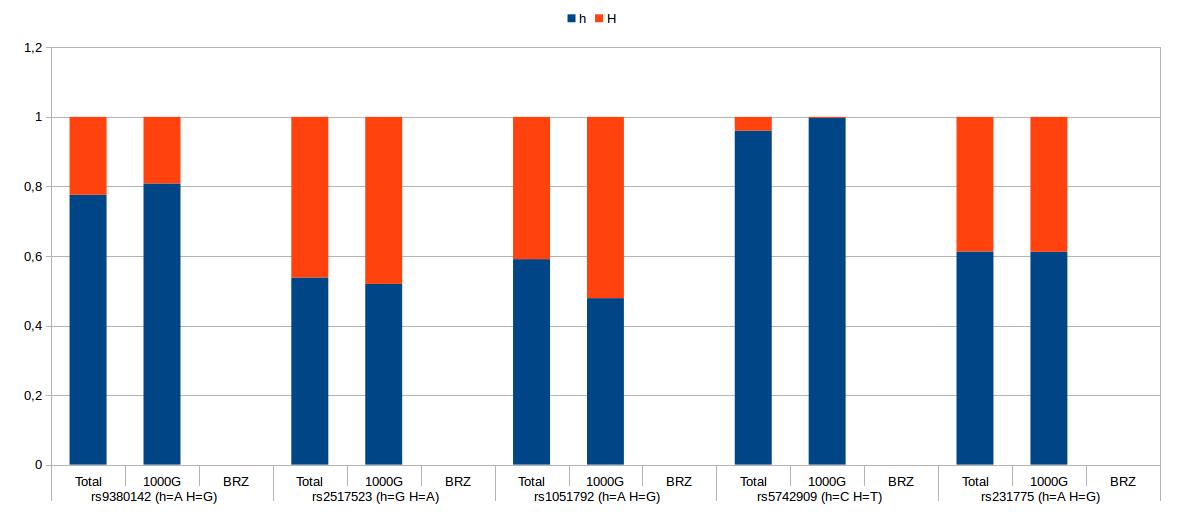

Supplement: Figure S2 — Comparison of the allele distribution of each SNP between our cohort and non-SCD populations. 1000G, 1,000 genomes database; BRZ, Brazilian non-SCD population. [file Image_6.jpg]
